# Supplementary figures and images for: Evaluation of mitochondrial DNA copy number estimation techniques
Source: PLoS One. 2020 Jan 31;15(1):e0228166. doi: 10.1371/journal.pone.0228166 (PMC6994099; doi:10.1371/journal.pone.0228166)

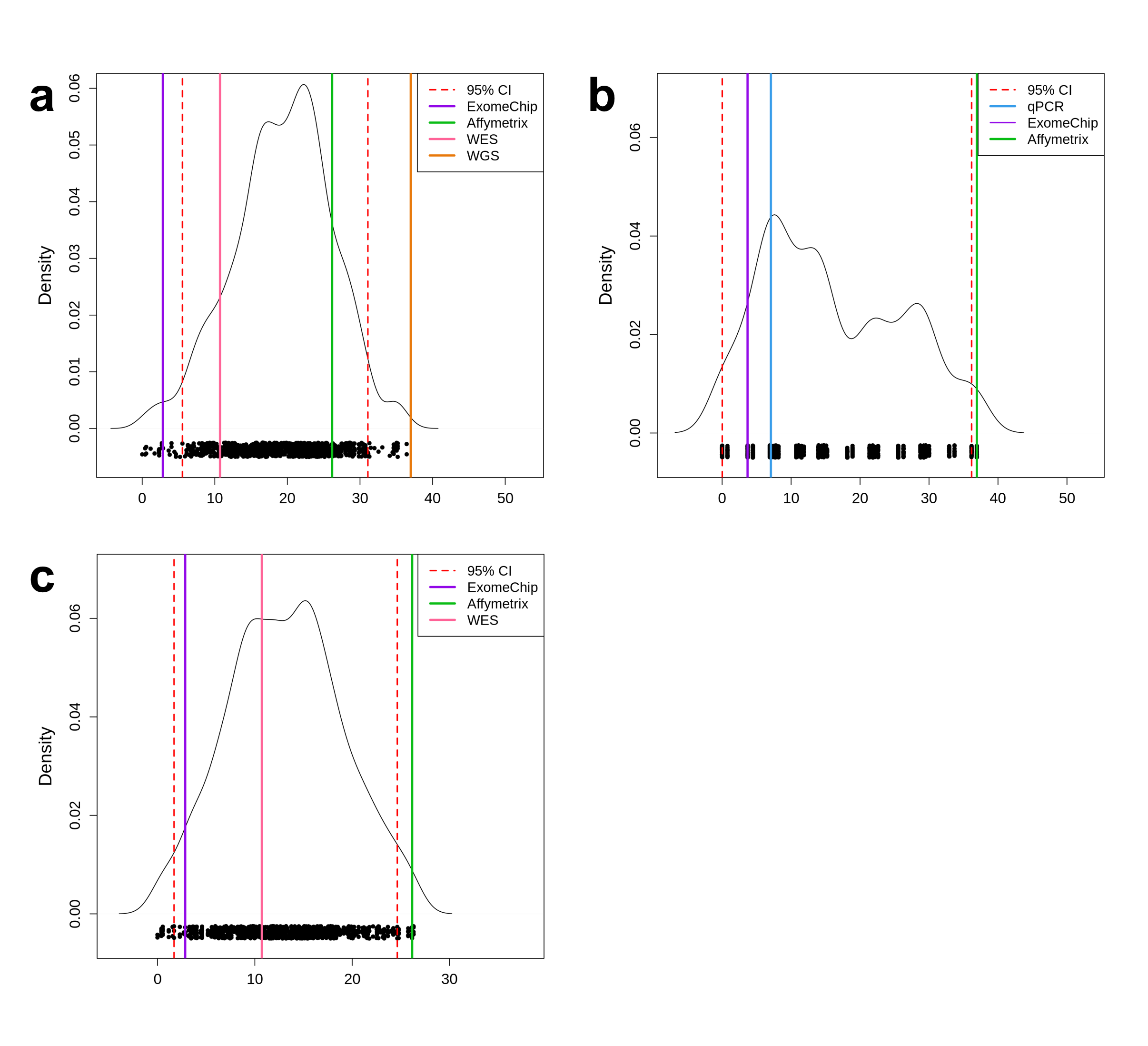

Supplement: S1 Fig — (TIF) [file pone.0228166.s001.tif]

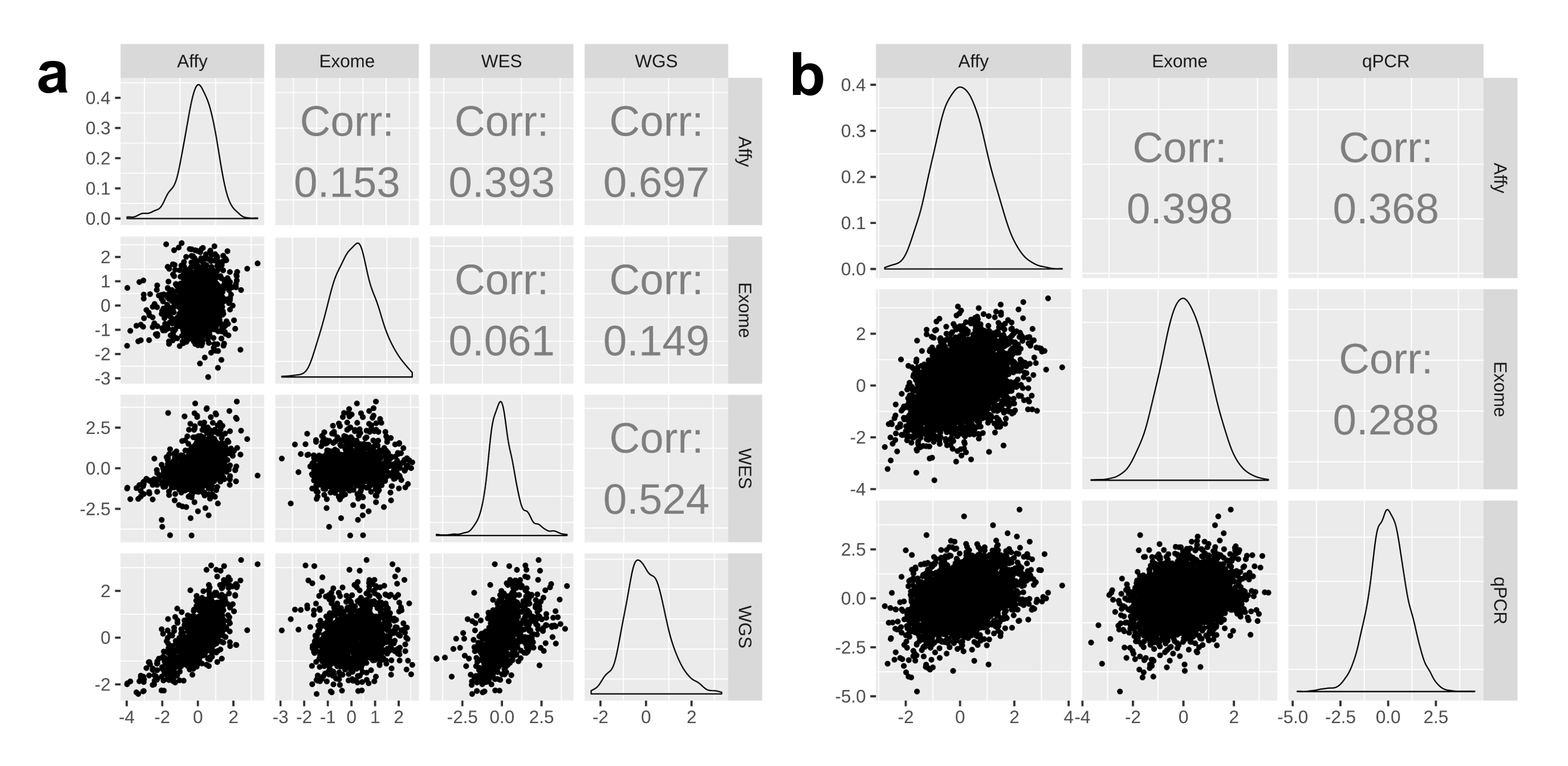

Supplement: S2 Fig — (TIF) [file pone.0228166.s002.tif]
