# Supplementary material for: Evaluation of mitochondrial DNA copy number estimation techniques
Source: PLoS One. 2020 Jan 31;15(1):e0228166. doi: 10.1371/journal.pone.0228166 (PMC6994099; doi:10.1371/journal.pone.0228166)
Supplement: Attachment — Submitted filename: Response To Reviewers.docx [file pone.0228166.s010.docx]

**Johns Hopkins University School of Medicine**

733 North Broadway

MRB Room 459

Baltimore, MD 21205

(410) 502-4867/ FAX (410) 614-8600

Email: arking@jhmi.edu

December 15^th^, 2019

We wish to thank the reviewer for their thorough and helpful review of our manuscript entitled **“****Evaluation of mitochondrial DNA copy number estimation techniques”**. We have addressed the reviewer comments in the revised manuscript and listed our responses to the comments below. Reviewer comments are listed in red text, our response is shown in black and text which was added to our manuscript is italicized in quote blocks.

**Comments:**

1. Can the authors please either (1) provide and explanation for why the Duffy locus was only analyzed in African Americans, or (2) provide citation(s) as justification?

We agree with the reviewer that we were not clear as to why the Duffy locus was 1) chosen as a correlate and 2) why it was only analyzed in Blacks. We have provided the following additional information between lines 259 and 263 on page 13 within the statistical analyses section of the Methods.

*“The Duffy locus is highly associated with WBC count in Blacks^26^ due to its role in conferring a selective advantage to malaria, however this association is limited or absent in other ethnicities^27^. As such, single SNP regression for mtDNA-CN on the Duffy locus was limited to Blacks. Due to the association of mtDNA-CN with WBC count, the Duffy locus acts as another independent external validator for mtDNA-CN unadjusted for WBC count.”*

2. Figure 3- the legend is seemingly mislabeled; I believe that the green series represents the ExomeChip data and the Purple series represents Affy.

Thank you, the figure has been modified.

3. Given that the authors only tested the “Lyse” method on cultured cells, which likely have fewer inhibitors compared to other sample types (e.g., heme carry over in blood derived samples), the authors should discuss briefly that the method may have limited applicability based on sample type.
We have added the following comment on page 19 (lines 416-419) within our discussion to highlight that our Lyse method needs further validation in non-cultured cells.

*“Additionally, as our application of the lyse method was limited to cultured cells, it is important to further validate this method in the context of different sample types which may have higher concentrations of inhibitors.”*

4. The authors discuss and evaluate relative qPCR for quantification of mtDNA-CN, but do not evaluate or discuss any of the published absolute qPCR assays; this may warrant a brief discussion point since absolute qPCR has the advantage of monitoring PCR efficiency (which can greatly alter copy number estimates) and batch effects.
We agree and have added a comment on page 19 of the discussion (lines 404-407) pointing out that absolute qPCR may improve upon the performance of mtDNA-CN estimation.

*“Furthermore, absolute quantification of mtDNA-CN through the use of standard curves may improve upon the performance of qPCR furthering its continuing use^37^.”*

5. Throughout the manuscript, the authors interchangeably use "black" and "African Americans"; I would suggest picking one or the other to maintain consistency.

Thank you, this change has been made as suggested

6. If the authors choose to use "white" and "black" as their racial descriptors, please capitalize the first letter of each term.

Thank you, we have capitalized all racial descriptors.

7. Pg.10: "DNA for mtDNA-CN estimation was collected from different visits and was derived from buffy coat using the Gentra Puregene Blood Kit (Qiagen)."---Can the authors verify that relevant variables (e.g., WBC count) were also collected from the same visit as the DNA sample?
We have clarified that relevant covariates were derived from the same visit in which the DNA was collected.

8. Pg.16: "Follow-up time was defined from DNA collection through death, loss to follow-up, or study end point (through 2017 in ARIC and 2015 in MESA)."—suggested edit to “lost to follow-up”.

The suggested edits have been made.

9. Many of the tables have poor resolution or illegible text; particularly, in supplemental Figure 2, the authors should consider changing the x and y axis labels to a larger font size.
Thank you for pointing this out, the figures and tables have been adjusted.

During the review process we were additionally made aware that the DNA used in our dPCR exploratory study was derived from packed red blood cells and not peripheral leukocytes as we previously believed. We have made this clear on page 6 of the methods as well as commented briefly on this difference on page 18 of the discussion the discussion.

*“Additionally, whereas the dPCR data was derived from DNA from packed red blood cells, the qPCR and Affymetrix data was obtained from peripheral leukocytes potentially explaining the poor performance of dPCR relative to other methods.”*

We thank you for your time and consideration of our revised manuscript. We look forward to your feedback.

Yours sincerely,

Dan E. Arking, PhD

Johns Hopkins University School of Medicine

733 N. Broadway

Miller Research Building, Room 459

Baltimore, MD 21205

[arking@jhmi.edu](mailto:arking@jhmi.edu)
